# Comprehensive analysis of the association between inflammation indexes and complications in patients undergoing pancreaticoduodenectomy

**DOI:** 10.3389/fimmu.2023.1303283

**Published:** 2023-11-23

**Authors:** Minghua Ma, Guo Li, Baoyong Zhou, Kaili Li, Zhongjun Wu, Lingwang Kong, Maoyun Liu, Miao Liu, Cheng Zhang, Huarong Yu, Shuaiqi Wang, Zuotian Huang, Kezhen Zong

**Affiliations:** ^1^ Department of Hepatobiliary Surgery, The First Affiliated Hospital of Chongqing Medical University, Chongqing, China; ^2^ Chongqing Medical University, Chongqing, China; ^3^ Chongqing University Cancer Hospital, Chongqing, China

**Keywords:** pancreaticoduodenectomy, blood routine examination, inflammatory indexes, restricted cubic splines (RCS), complication

## Abstract

**Background:**

During clinical practice, routine blood tests are commonly performed following pancreaticoduodenectomy (PD). However, the relationship between blood cell counts, inflammation-related indices, and postoperative complications remains unclear.

**Method:**

We conducted a retrospective study, including patients who underwent PD from October 2018 to July 2023 at the First Hospital of Chongqing Medical University, and compared baseline characteristics and clinical outcomes among different groups. Neutrophil count (NC), platelet count (PLT), lymphocyte count (LC), systemic immune-inflammation index (SII), platelet-to-lymphocyte ratio (PLR), neutrophil-to-lymphocyte ratio (NLR), and the product of platelet count and neutrophil count (PPN) were derived from postoperative blood test results. We investigated the association between these indicators and outcomes using multivariable logistic regression and restricted cubic spline analysis. The predictive performance of these indicators was assessed by the area under the curve (AUC) of the receiver operating characteristic (ROC) curve and decision curve analysis (DCA).

**Result:**

A total of 232 patients were included in this study. Multivariate logistic regression and restricted cubic spline analysis showed that all indicators, except for PLT, were associated with clinical postoperative pancreatic fistula (POPF). SII, NLR, and NC were linked to surgical site infection (SSI), while SII, NLR, and PLR were correlated with CD3 complication. PLT levels were related to postoperative hemorrhage. SII (AUC: 0.729), NLR (AUC: 0.713), and NC (AUC: 0.706) effectively predicted clinical POPF.

**Conclusion:**

In patients undergoing PD, postoperative inflammation-related indices and blood cell counts are associated with various complications. NLR and PLT can serve as primary indicators post-surgery for monitoring complications.

## Introduction

Pancreaticoduodenectomy (PD) represents a crucial therapeutic intervention for conditions affecting the pancreatic head, duodenum, and bile duct. However, owing to its intricate nature, the emergence of postoperative complications remains a formidable challenge in PD cases. A recently published systematic review encompassing 63,229 PD procedures revealed that mortality, morbidity, and the occurrence of severe complications constituted 1.7%, 54.7%, and 25.5% of patients post-PD, respectively (1). These postoperative complications not only extend the duration of hospital stays but also exert a significant impact on patient prognosis (2, 3). Simultaneously, the high incidence of postoperative complications contributes significantly to the escalation of healthcare costs (4, 5). Thus, the early identification of patients at heightened risk of postoperative complications is essential.

The complete blood count (CBC) test, characterized by its simplicity and routine nature, involves the collection of small blood samples from patients, facilitating the assessment of their current health status by clinicians. This test yields a plethora of blood cell counts, including neutrophil count (NC), platelet count (PLT), and lymphocyte count (LC). From these counts, an array of inflammation-related indices, such as the systemic immune-inflammation index (SII), platelet-to-lymphocyte ratio (PLR), neutrophil-to-lymphocyte ratio (NLR), and the product of platelet count and neutrophil count (PPN), can be derived (6). Over the years, numerous studies have revealed the association between inflammation-related indices and a wide spectrum of diseases, encompassing neoplastic conditions, autoimmune disorders, metabolic maladies, neuropsychiatric disorders, cardiovascular ailments, and infectious diseases (7–17). In this respect, SII has emerged as a prognostic marker following PD in patients with neoplastic diseases (18, 19). NLR has also been found to be associated with postoperative complications in PD (20, 21). However, there is still a lack of systematic analysis and comparison of the inflammatory indices in PD. Despite the ubiquity of routine blood tests such as CBC as a postoperative examination, the relationship between inflammation-related indices derived from CBC tests and postoperative complications in PD has been largely understudied. This study aims to address two key inquiries: firstly, the identification of inflammation-related indicators from the CBC that are linked to postoperative outcomes in patients with PD; and secondly, the determination of specific inflammation-related indicators that should be prioritized in postoperative monitoring. By examining the relationship between inflammation-related indices and postoperative complications, this research endeavors to offer significant evidence for enhancing clinical management, utilizing the results obtained from postoperative CBC analysis in PD patients.

## Method

### Study population and data collection

The study cohort comprised consecutive PD cases from October 2018 to July 2023, sourced from the First Hospital of Chongqing Medical University. Ethical approval for this study was granted under reference number K2023-332, as approved by the Ethics Committee of the First Hospital of Chongqing Medical University. Data encompassing patient demographics, medical records, laboratory analyses, imaging studies, and surgical records of PD patients were retrospectively collected through the electronic medical record system. Exclusion criteria encompassed non-PD procedures, autoimmune and hematologic disorders, preoperative infectious complications, involvement of non-hepatobiliary and pancreatic surgeons, multiorgan surgeries, robotic procedures, incomplete data, and patients below the age of 18 years. Patients without postoperative day 3 (POD3) results of blood routine tests were excluded but included in sensitivity analyses.

### Surgical procedure and perioperative management

Patients who underwent PD within the past five years were included in the study. All PD procedures were executed by experienced surgeons, with both open and laparoscopic classical Whipple procedures performed at our institution. Duct-to-mucosa anastomosis was the predominant technique for pancreaticojejunostomy. Intraperitoneal drains were routinely placed during surgery to monitor postoperative pancreatic fistula (POPF). Prophylactic antibiotics were administered to all patients, with an extended antibiotic regimen postoperatively. Somatostatin or octreotide treatment was usually applied until 5 to 7 days postoperatively. Acid suppressants were continued for several months following the procedure. Patients initially received parenteral nutrition in the early postoperative phase and were gradually transitioned to enteral nutrition. Regular abdominal imaging studies were conducted postoperatively.

### Definition and outcomes

In this study, inflammation-related indices and blood cell counts were derived from complete blood count test results obtained on POD3. The systemic immune-inflammation index (SII) was calculated as the product of PLT multiplied by NC and then divided by LC. The neutrophil-to-lymphocyte ratio (NLR) was calculated as NC divided by LC, while the platelet-to-lymphocyte ratio (PLR) was computed as PLT divided by LC. Additionally, the product of platelet count and neutrophil count (PPN) was obtained by multiplying PLT by NC.

The primary outcome of interest in this study was clinical POPF, which was diagnosed and classified according to the International Study Group for Pancreatic Surgery (ISGPS) criteria (22). Grade B and C POPF cases were collectively defined as clinical POPF. Secondary outcomes included surgical site infection (SSI), intra-abdominal hemorrhage, Clavien-Dindo grade III (CD3) complications (inclusive of those greater than grade III), reoperation, and readmission. Readmission was defined as unplanned readmission with an electronic record. Diagnoses other than pancreatic cancer and chronic pancreatitis were high-risk diagnoses. Other outcome definitions were consistent with the literature (23).

### Statistical analysis

The study cohort was categorized into two groups based on the 75th percentile of POD3 SII values, resulting in a low SII group and a high SII group. Baseline characteristics and clinical outcomes were compared between these two groups of PD patients. Continuous variables were reported as medians with interquartile ranges (IQR) or means with standard deviations (SD) and compared using the Wilcoxon rank-sum test or student t-test, as appropriate. Categorical variables were presented as counts with percentages (%) and compared using Fisher’s exact test or Pearson’s Chi-squared test, as appropriate.

Logistic regression analysis was conducted to estimate odds ratios (OR) and 95% confidence intervals (CI) to elucidate the association between POD3 inflammation-related indices (including related blood cell counts) and various outcomes. To facilitate the presentation of estimated ORs, some inflammation-related indices and blood cell counts were standardized. Three models were constructed with different levels of adjustment. Model 1 did not include any adjustments, while Model 2 incorporated adjustments for patients’ general characteristics, including age, gender, body mass index (BMI), high-risk diagnosis, albumin levels, diabetes mellitus, and baseline levels of relevant exposure factors. Model 3 built upon Model 2 by further adjusting for characteristics related to pancreatic surgery, such as preoperative biliary drainage (PBD), main pancreatic duct diameter (MPD), surgical approach, operative time, and intraoperative hemorrhage volume. Logistic regression analysis was repeated after transforming inflammation-related indices and blood cell counts from continuous to categorical variables based on quartiles and medians as part of sensitivity analysis.

To visualize the linear or non-linear relationship between inflammation-related indices, blood cell counts, and different complications following pancreaticoduodenectomy, multivariable restricted cubic spline (RCS) analyses based on logistic models were conducted. These models included covariates such as age, gender, BMI, pathological diagnosis, albumin levels, diabetes mellitus, and baseline levels of relevant exposure factors. At the 10th, 50th, and 90th percentiles, three knots were employed to construct the models (24, 25). Patients initially excluded due to the absence of POD3 routine blood test results were reintegrated, utilizing results from POD2, POD4, or POD5 blood routine tests as part of sensitivity analysis. Receiver operating characteristic (ROC) curves were generated to calculate the area under the curve (AUC), evaluating the predictive capability of inflammation-related indices and blood cell counts for different outcomes. Additionally, decision curve analysis (DCA) was performed. A significance threshold of *P* < 0.05 was set for statistical significance. All statistical analyses were carried out using R 4.2.0, utilizing various R packages, including ‘gtsummary’, ‘rcssci’, ‘pROC’, ‘ggDCA’, and ‘stats’.

## Results

### Study population characteristics and outcomes

**Figure 1:**
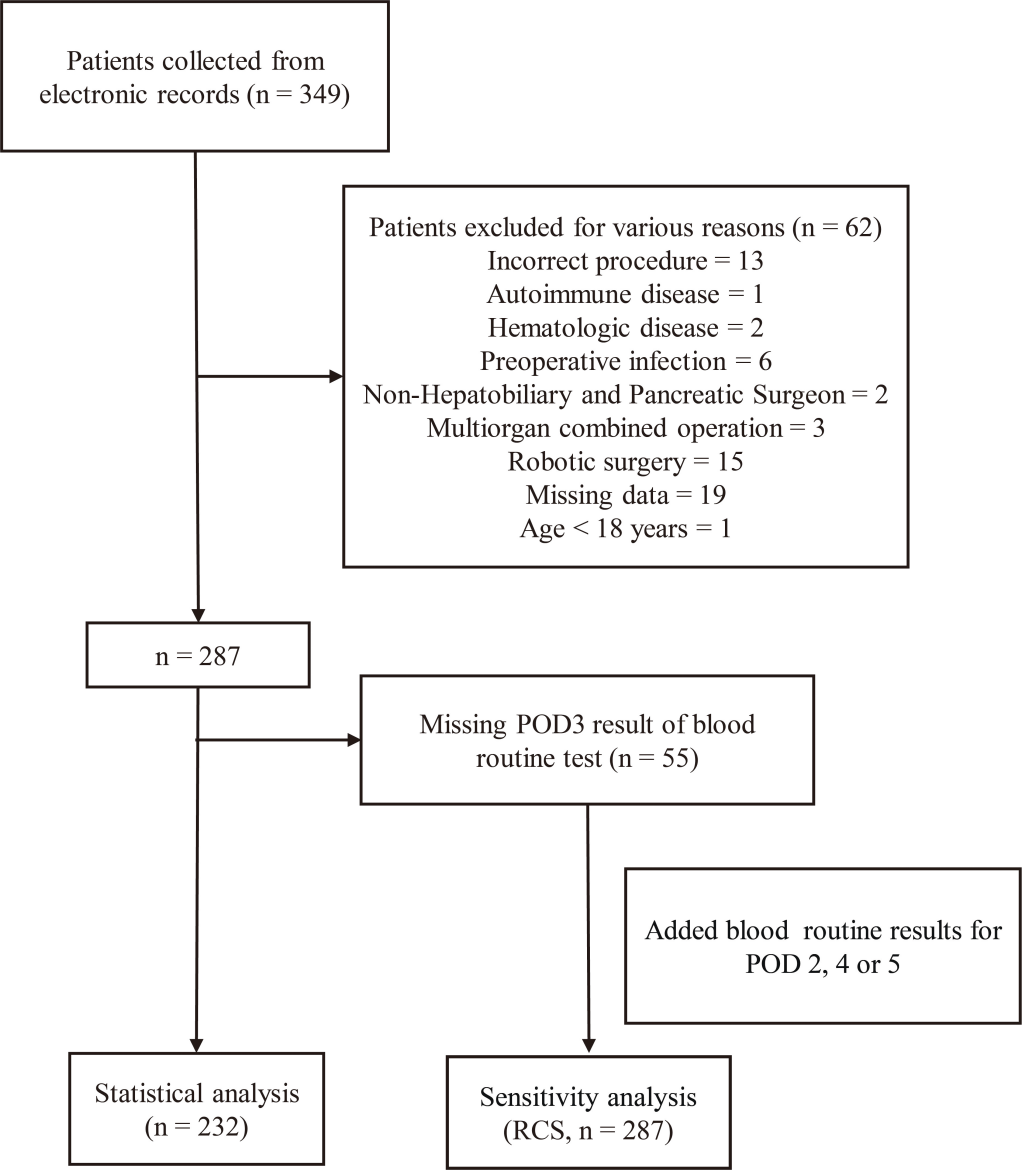
The flowchart of inclusion of patients. POD3, postoperative day 3.

A total of 349 patients with complete medical records were included, with 117 patients excluded, resulting in the inclusion of 232 patients in the study. The inclusion process is illustrated in **Figure 1**. The cohort was stratified into a low SII group (n=174) and a high SII group (n=58) based on the third quartile of POD3 SII (2783). No significant differences were observed in age, BMI, hypertension, other cancer diagnoses, abdominal surgery history, smoking, or alcohol consumption between the two groups. However, gender and diabetes mellitus distribution differed significantly (*P* < 0.05). Significant differences were also found in PLT and albumin levels in the preoperative tests (*P* < 0.05). No disparities were noted in other blood cell counts or liver function indices. Furthermore, there were no discrepancies in terms of diagnosis, preoperative biliary drainage, main pancreatic duct diameter, prophylactic antibiotic use, surgical approach, intraoperative blood loss, or operative time. Specific patient characteristics are outlined in **Table 1**.

**Table 1:** The baseline characteristics of included patients.

Characteristic	Low-SII, N = 174	High-SII, N = 58	p-value
Age (years)	61 (10)	61 (11)	0.938
Gender			0.039
Female	55 (32%)	27 (47%)	
Male	119 (68%)	31 (53%)	
BMI (kg/m²)	22.5 (3.4)	21.9 (2.6)	0.125
Diabetes mellitus	34 (20%)	3 (5.2%)	0.010
Hypertension	33 (19%)	12 (21%)	0.774
Other cancers	10 (5.7%)	1 (1.7%)	0.300
Abdominal operation history	38 (22%)	13 (22%)	0.927
Smoke	72 (41%)	21 (36%)	0.486
Drink	51 (29%)	15 (26%)	0.614
Platelet count (10^9^/L)	198 (160, 255)	253 (215, 302)	<0.001
Neutrophil count (10^9^/L)	3.72 (2.92, 4.63)	3.99 (3.11, 5.52)	0.162
Lymphocyte count (10^9^/L)	1.17 (0.96, 1.50)	1.09 (0.90, 1.42)	0.170
Albumin (g/L)	40.0 (36.0, 43.0)	37.0 (34.0, 41.8)	0.004
Bilirubin (ummol/L)	98 (14, 191)	81 (17, 167)	0.702
ALT(U/L)	142 (34, 229)	132 (63, 209)	0.990
AST(U/L)	89 (30, 187)	94 (60, 168)	0.596
High risk diagnosis	88 (51%)	33 (57%)	0.404
Pathologic diagnosis			0.054
bile duct cancer	27 (16%)	13 (22%)	
duodenal/ampullary cancer	32 (18%)	15 (26%)	
IPMN	9 (5.2%)	0 (0%)	
NET	0 (0%)	1 (1.7%)	
other	20 (11%)	4 (6.9%)	
pancreatic cancer	77 (44%)	25 (43%)	
pancreatitis	9 (5.2%)	0 (0%)	
PBD	61 (35%)	18 (31%)	0.576
MPD < 3mm	52 (30%)	22 (38%)	0.255
Prophylactic antibiotics			0.128
Broad-abx	33 (19%)	6 (10%)	
Narrow-abx	141 (81%)	52 (90%)	
Approach			0.356
LPD	99 (57%)	37 (64%)	
OPD	75 (43%)	21 (36%)	
Intraoperative blood loss (ml)	300 (200, 400)	200 (150, 300)	0.056
Operative time (min)	415 (350, 478)	390 (351, 440)	0.152

BMI, body mass index; ALT, alanine aminotransferase; AST, aspartate aminotransferase; IPMN, intraductal papillary mucinous neoplasms; NET, neuroendocrine tumor; PBD, preoperative biliary drainage; MPD, main pancreatic duct diameter; Broad-abx, broad-spectrum antibiotic; Narrow-abx, narrow-spectrum antibiotic; LPD, laparoscopic pancreaticoduodenectomy; OPD, open pancreaticoduodenectomy; High risk diagnosis, diagnosis beyond pancreatitis and pancreatic cancer.

**Table 2** presents the postoperative test results and outcomes for both groups. Significant differences were observed in POD3 inflammation-related indices and blood cell count between the two groups (*P* < 0.05), with the differences consistent with SII. Nevertheless, among all the outcomes, differences between the two groups were only significant in clinical POPF, suggesting a potential association between SII and other inflammation-related indices with clinical POPF. However, investigating the association between inflammation-related indices and postoperative outcomes of PD necessitates the consideration of potential confounding factors.

**Table 2:** The outcome of included patients.

Outcomes	Low-SII, N = 174	High-SII, N = 58	p-value
POD3 NC (10^9^/L)	8.0 (6.3, 10.3)	12.2 (9.9, 14.9)	<0.001
POD3 PLT (10^9^/L)	144 (114, 191)	217 (158, 252)	<0.001
POD3 LC (10^9^/L)	0.77 (0.59, 1.01)	0.60 (0.42, 0.81)	<0.001
POD3 PLR	195 (140, 247)	352 (303, 446)	<0.001
POD3 NLR	10 (8, 14)	19 (15, 27)	<0.001
POD3 PPN	1,107 (842, 1,752)	2,703 (1,749, 3,549)	<0.001
B/C POPF	37 (21%)	27 (47%)	<0.001
SSI	33 (19%)	16 (28%)	0.164
Hemorrhage	25 (14%)	9 (16%)	0.830
Re-operation	13 (7.5%)	5 (8.6%)	0.780
Readmission	15 (8.6%)	5 (8.6%)	>0.999
CD3 complication	27 (16%)	12 (21%)	0.362
Death	6 (3.4%)	2 (3.4%)	>0.999

SII, systemic immune-inflammation index; POD3, postoperative day 3; NC, neutrophil count; PLT, platelet; LC, lymphocyte count; PLR, platelet-to-lymphocyte ratio; NLR, neutrophil-to-lymphocyte ratio; PPN, the product of platelet count and neutrophil count; POPF, postoperative pancreatic fistula; SSI, surgical site infection; CD3, Clavien-Dindo grade III.

### The association of inflammation-related indexes with outcomes

#### Clinical POPF

**Figure 2:**
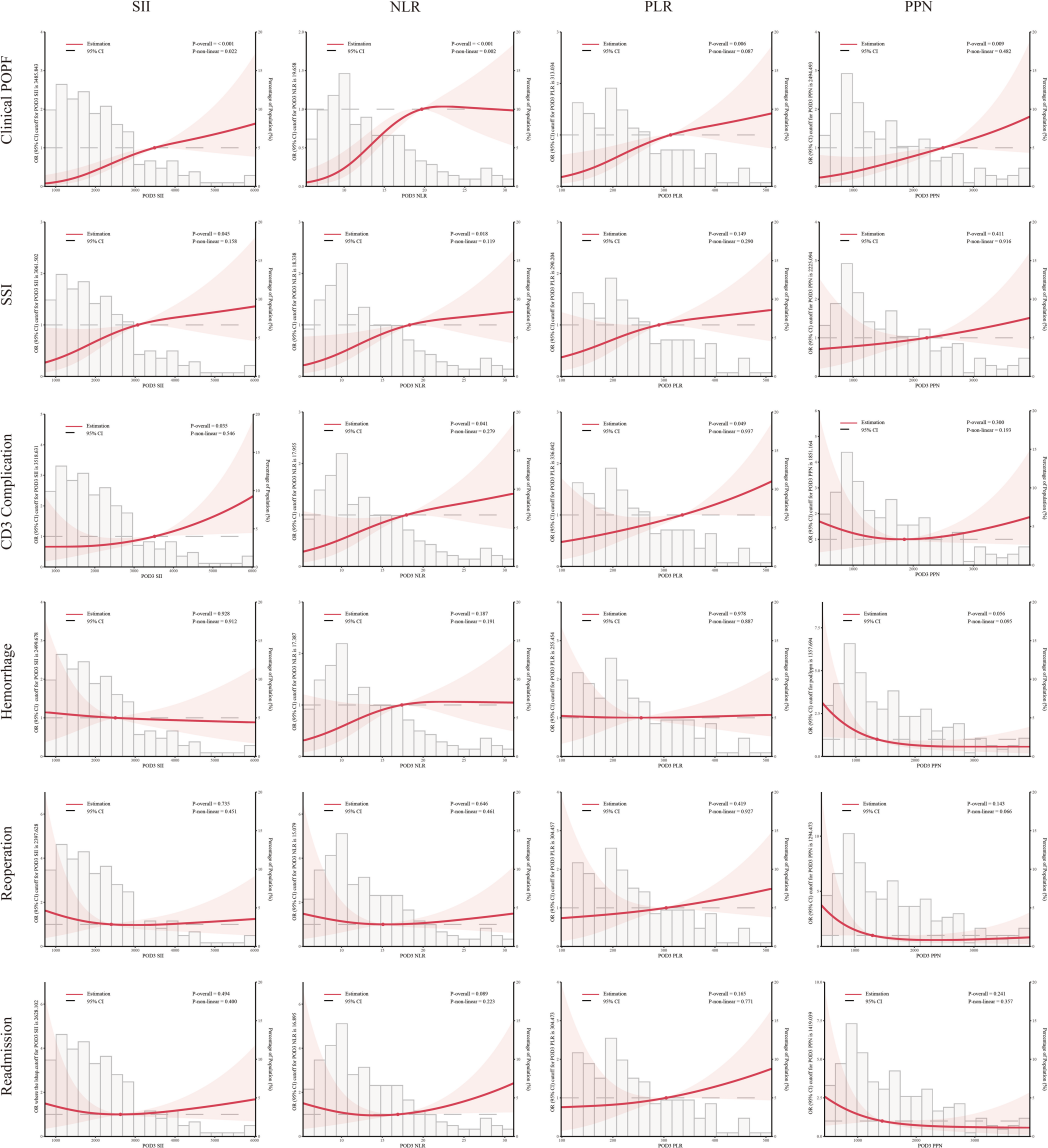
Multivariable restricted cubic spline of inflammation-related indexes. The threshold of significance is *P*-overall < 0.05. The model was conducted with 3 knots at the 10th, 50th, 90th percentiles. Solid lines indicate ORs, and shadow shape indicate 95% CIs. Systemic immune-inflammation index (SII), platelet-to-lymphocyte ratio (PLR), neutrophil-to-lymphocyte ratio (NLR) and the product of platelet count and neutrophil count (PPN).

**Figure 3:**
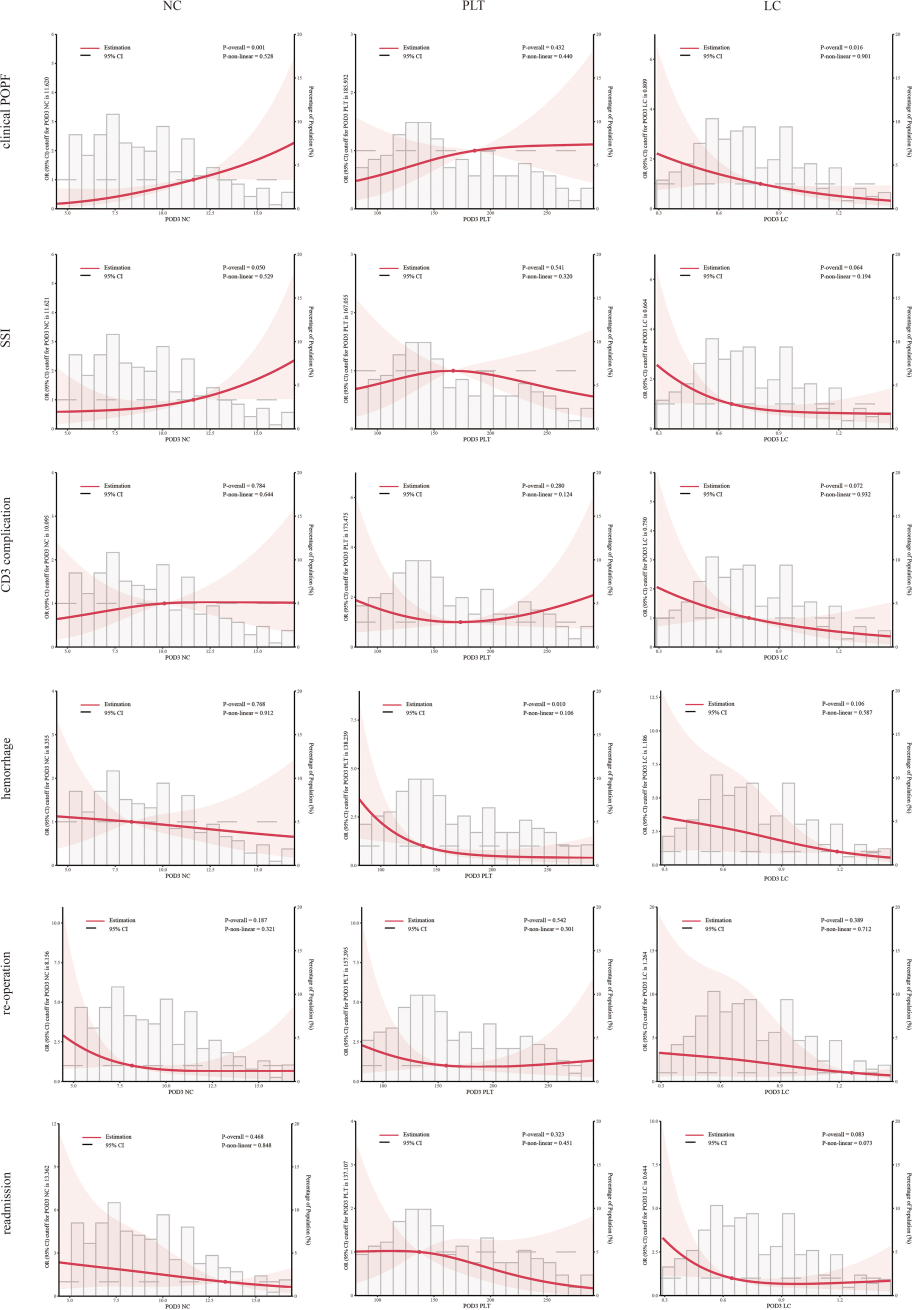
Multivariable restricted cubic spline of blood cell count. The threshold of significance is *P*-overall < 0.05. The model was conducted with 3 knots at the 10th, 50th, and 90th percentiles. Solid lines indicate ORs, and shadow shapes indicate 95% CIs. Neutrophil count (NC), platelet (PLT), and lymphocyte count (LC).

SII, NLR, PLR, PPN, and NC consistently exhibited significant correlations with clinical POPF during regression analyses across the three models (**Table 3**). During sensitivity analyses, when treated as categorical variables, patients with higher SII, NLR, PLR, PPN, and NC demonstrated a significantly elevated risk of clinical POPF across all three models (**Table S1**). After visualizing the linear or non-linear relationship through RCS, the aforementioned indicators exhibited a significant positive correlation with the risk of clinical POPF (*P-overall* < 0.05, **Figures 2**, **3**), a finding consistent with the results of sensitivity analysis upon reintegrating excluded patients (*P-overall* < 0.05, **Figures S1**, **S2**). In our cohort, the significant cutoff values for each indicator were as follows: SII 3485.843, NLR 19.658, PLR 313.034, PPN 2494.493, and NC 11.620. The results from the three models did not consistently suggest an association between PLT and clinical POPF (**Tables 3**, **S1**). The significant correlation between PLT and clinical POPF was not supported by RCS (**Figures 3**, **S2**). Additionally, while not significant as a categorical variable, as a continuous variable, a lower LC implied a higher risk of POPF (**Table 3**, model 2: OR 0.86, 95%CI 0.77-0.95, *P*=0.005; model 3: OR 0.87, 95%CI 0.77-0.97, *P*=0.016). Further analyses also suggested a significant correlation (**Figures 3**, **S2**, *P-overall* < 0.05, cutoff value = 0.809).

**Table 3:** Association with clinical POPF, SSI and CD3 complication.

Indexes	Model 1			Model 2			Model 3		
	OR	95% CI	p-value	OR	95% CI	p-value	OR	95% CI	p-value
Clinical POPF									
SII^a^	1.04	1.02, 1.07	<0.001	1.04	1.02, 1.07	<0.001	1.05	1.02, 1.07	<0.001
NLR	1.08	1.04, 1.12	<0.001	1.07	1.03, 1.12	<0.001	1.07	1.02, 1.11	0.003
PLR^b^	1.03	1.01, 1.05	0.007	1.04	1.01, 1.07	0.005	1.04	1.01, 1.07	0.009
PPN^c^	1.06	1.03, 1.09	<0.001	1.05	1.02, 1.09	0.004	1.06	1.02, 1.10	0.004
NC	1.23	1.13, 1.34	<0.001	1.19	1.08, 1.32	<0.001	1.18	1.06, 1.31	0.002
PLT^d^	1.05	1.00, 1.09	0.037	1.03	0.98, 1.10	0.300	1.05	0.99, 1.13	0.120
LC^e^	0.94	0.86, 1.02	0.200	0.86	0.77, 0.95	0.005	0.87	0.77, 0.97	0.016
Surgical site infection									
SII^a^	1.02	1.01, 1.04	0.012	1.02	1.00, 1.04	0.031	1.02	1.00, 1.04	0.075
NLR	1.05	1.02, 1.09	0.005	1.05	1.01, 1.09	0.010	1.05	1.01, 1.10	0.023
PLR^b^	1.02	1.00, 1.04	0.07	1.02	1.00, 1.05	0.044	1.03	1.00, 1.06	0.057
PPN^c^	1.03	1.01, 1.06	0.018	1.02	0.99, 1.05	0.300	1.02	0.98, 1.05	0.400
NC	1.13	1.04, 1.24	0.004	1.10	1.00, 1.22	0.053	1.09	0.98, 1.22	0.110
PLT^d^	1.01	0.97, 1.06	0.500	1.03	0.98, 1.10	0.300	0.98	0.92, 1.05	0.600
LC^e^	0.98	0.89, 1.07	0.600	0.90	0.80, 0.99	0.049	0.88	0.77, 0.99	0.037
CD3 complication									
SII^a^	1.02	1.00, 1.04	0.027	1.02	1.01, 1.05	0.014	1.03	1.01, 1.05	0.009
NLR	1.04	1.00, 1.08	0.028	1.05	1.01, 1.09	0.018	1.05	1.01, 1.10	0.013
PLR^b^	1.02	1.00, 1.04	0.053	1.03	1.01, 1.05	0.014	1.04	1.01, 1.07	0.006
PPN^c^	1.01	0.98, 1.04	0.3	1.01	0.97, 1.04	0.600	1.01	0.97, 1.05	0.600
NC	1.04	0.94, 1.14	0.4	1.01	0.91, 1.13	0.800	1	0.89, 1.12	>0.900
PLT^d^	1.01	0.95, 1.06	0.8	1.00	0.94, 1.08	>0.900	1.02	0.94, 1.09	0.700
LC^e^	0.92	0.82, 1.02	0.14	0.86	0.75, 0.97	0.018	0.83	0.71, 0.94	0.007

SII, systemic immune-inflammation index; NLR, neutrophil-to-lymphocyte ratio; PLR, platelet-to-lymphocyte ratio; PPN, the product of platelet count and neutrophil count; NC, neutrophil count; PLT, platelet; LC, platelet: lymphocyte count; OR, odds ratio; CI, confidence interval.

a: SII/100; b: PLR/10; c: PPN/10; d: PLT/10; e: LC*10

Model 1: unadjusted.

Model 2: adjusted for age, gender, body mass index, high-risk diagnosis, albumin, diabetes mellitus and baseline level of related exposure factor.

Model 3: model 2 + further adjusted for preoperative biliary drainage (PBD), main pancreatic duct diameter (MPD), surgical approach, operative time and volume of intraoperative hemorrhage.

#### SSI

Logistic regression analyses indicated that elevated SII and NLR were associated with an increased risk of SSI (**Table 3**), consistent with the RCS results (**Figures 2**, **S1**). However, the results from model 3 did not support an association between PLR and SSI (**Table 3**, OR: 1.03, 95%CI:1.00-1.06, *P*=0.057). During sensitivity analyses, when assessed as a categorical variable, PLR was also not associated with SSI in all three models (**Table S2**, Q4: model 1-3, *P_1_ =* 0.2, *P_2_ =* 0.058, *P_3_ =* 0.054). Furthermore, RCS failed to demonstrate a significant alteration in the risk of SSI with changes in PLR (**Figures 2**, **S1**, *P-overall >*0.05). Analyses did not indicate any association between PPN and SSI. The primary results also failed to substantiate the relationship between PLT and SSI (**Figures 3**, **S2**; **Table 3**). The regression analysis results implied that lower LC (as a continuous variable) was associated with a higher risk of SSI (**Table 3**, model 2 and 3), in contrast to the sensitivity analyses (**Table S2**). This inconsistency was also observed in the RCS (**Figure 3**: *P-overall* = 0.064, **Figure S2**: *P-overall* = 0.017). The results of logistic regression were inconsistent with the RCS results when evaluating the relationship between NC and SSI.

#### CD3 complication

Logistic regression results showed that SSI, NLR, and PLR were associated with CD3 complications as continuous variables rather than categorical variables (**Tables 3**, **S3**). This inconsistency was also reflected in the RCS results (**Figures 2**, **S1**). PPN, NC, and PLT were not associated with CD3 complications. Logistic regression results indicated that LC was associated with CD3 complications as both continuous and categorical variables (**Table 3**: model 2 and 3, *P*<0.05; **Table S3**: Q4, model 3, *P*<0.05). However, the results from the RCS revealed no statistical significance (**Figure 3** *P-overall*=0.072, **Figure S2** *P-overall*=0.152).

#### Hemorrhage

Interestingly, we found that a lower PPN indicated a higher risk of postoperative hemorrhage (**Figure S2**; **Table S4**). However, this result was not confirmed by other analyses (**Figure 2**; **Table 4**). In both model 2 and model 3, PLT and LC demonstrated a significant correlation with postoperative hemorrhage (**Table 4**) when treated as continuous variables rather than categorical variables (**Table S4**). Nonetheless, the RCS results suggested that only PLT was associated with postoperative hemorrhage (**Figures 3**, **S2**).

**Table 4:** Association with clinical hemorrhage, reoperation and readmission.

Indexes	Model 1			Model 2			Model 3		
	OR	95% CI	p-value	OR	95% CI	p-value	OR	95% CI	p-value
Hemorrhage									
SII^a^	1.00	0.97, 1.02	0.800	1.00	0.97, 1.02	0.700	1.00	0.97, 1.03	0.800
NLR	1.03	0.99, 1.07	0.120	1.03	0.99, 1.07	0.200	1.04	1.00, 1.09	0.050
PLR^b^	1.00	0.97, 1.02	>0.900	1.00	0.97, 1.03	0.800	1.01	0.98, 1.04	0.400
PPN^c^	0.98	0.95, 1.02	0.400	0.96	0.91, 1.00	0.090	0.97	0.92, 1.01	0.200
NC	1.01	0.91, 1.11	0.900	0.96	0.85, 1.07	0.500	0.96	0.85, 1.09	0.600
PLT^d^	0.95	0.89, 1.00	0.077	0.90	0.83, 0.98	0.014	0.92	0.84, 1.0	0.044
LC^e^	0.91	0.80, 1.02	0.110	0.86	0.75, 0.98	0.027	0.82	0.70, 0.94	0.007
Reoperation									
SII^a^	1.00	0.97, 1.03	0.800	1.00	0.97, 1.03	0.900	1.01	0.97, 1.04	0.700
NLR	1.02	0.97, 1.07	0.400	1.02	0.96, 1.07	0.500	1.02	0.97, 1.08	0.400
PLR^b^	1.02	0.99, 1.04	0.200	1.02	0.99, 1.05	0.130	1.03	1.00, 1.07	0.027
PPN^c^	0.99	0.93, 1.03	0.600	0.97	0.90, 1.02	0.300	0.96	0.89, 1.02	0.200
NC	0.92	0.78, 1.05	0.200	0.86	0.71, 1.01	0.090	0.81	0.66, 0.98	0.044
PLT^d^	1.00	0.92, 1.07	>0.900	0.97	0.87, 1.06	0.500	0.97	0.87, 1.07	0.500
LC^e^	0.91	0.77, 1.06	0.300	0.86	0.71, 1.01	0.086	0.82	0.67, 0.97	0.030
Readmission									
SII^a^	1.01	0.98, 1.03	0.400	1.01	0.99, 1.04	0.300	1.02	0.99, 1.04	0.200
NLR	1.05	1.00, 1.09	0.037	1.05	1.00, 1.09	0.047	1.05	1.00, 1.11	0.042
PLR^b^	1.02	0.99, 1.04	0.200	1.03	1.00, 1.05	0.036	1.03	1.00, 1.06	0.021
PPN^c^	0.98	0.93, 1.02	0.500	0.96	0.90, 1.02	0.200	0.97	0.91, 1.02	0.300
NC	0.96	0.84, 1.09	0.600	0.90	0.76, 1.04	0.200	0.89	0.74, 1.04	0.200
PLT^d^	0.96	0.89, 1.03	0.300	0.95	0.85, 1.05	0.300	0.97	0.87, 1.07	0.500
LC^e^	0.92	0.79, 1.06	0.300	0.91	0.77, 1.05	0.200	0.90	0.74, 1.06	0.200

SII, systemic immune-inflammation index; NLR, neutrophil-to-lymphocyte ratio; PLR, platelet-to-lymphocyte ratio; PPN, the product of platelet count and neutrophil count; NC, neutrophil count; PLT, platelet; LC, platelet: lymphocyte count; OR, odds ratio; CI, confidence interval.

a: SII/100; b: PLR/10; c: PPN/10; d: PLT/10; e: LC*10.

Model 1: unadjusted.

Model 2: adjusted for age, gender, body mass index, high-risk diagnosis, albumin, diabetes mellitus and baseline level of related exposure factor.

Model 3: model 2 + further adjusted for preoperative biliary drainage (PBD), main pancreatic duct diameter (MPD), surgical approach, operative time and volume of intraoperative hemorrhage.

#### Reoperation

During regression analyses, an association with reoperation was only observed in model 3 when PLR (OR: 1.03, 95%CI:1.00-1.07, *P*=0.027), NC (OR: 0.81, 95%CI: 0.66-0.98, *P*=0.044), and LC (OR: 0.82, 95%CI: 0.67-0.97, *P*=0.03) were used as continuous variables (**Table 4**). The remaining results did not indicate any correlation.

#### Readmission

NLR (model 1-3, *P*<0.05) and PLR (model 2 and 3, *P*<0.05) were potentially associated with an increased risk of readmission when assessed as continuous variables (**Table 4**). However, RCS analysis did not confirm this correlation (**Figures 2**, **S1**).

#### Predictive ability of indicators

**Figure 4:**
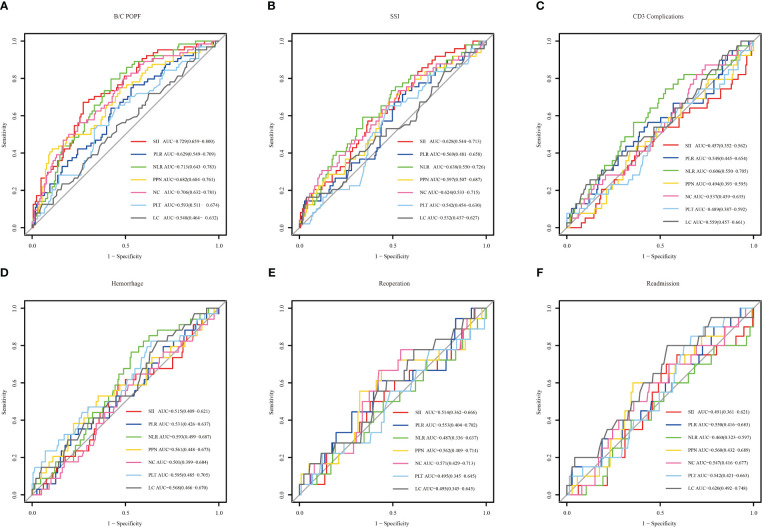
The receiver operating characteristic of inflammation-related indexes and blood cell counts for different outcomes. Systemic immune-inflammation index (SII), platelet-to-lymphocyte ratio (PLR), neutrophil-to-lymphocyte ratio (NLR), and the product of platelet count, neutrophil count (PPN), neutrophil count (NC), platelet (PLT) and lymphocyte count (LC). **(A–F)** represent clinical POPF, SSI, CD3 complications, hemorrhage, reoperation, and readmission, respectively.

**Figure 5:**
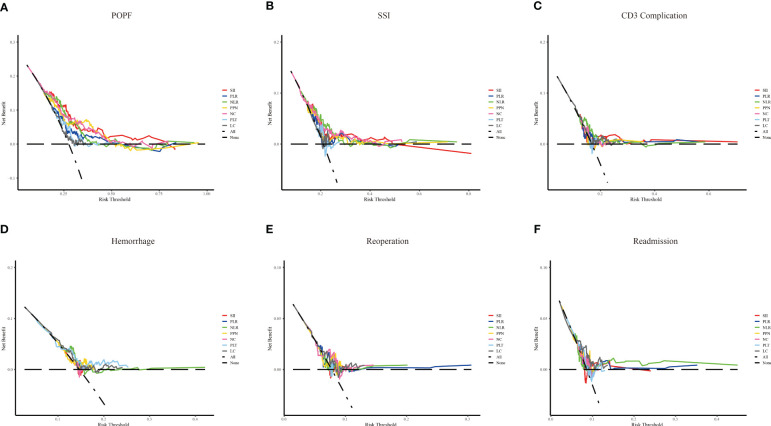
The decision curve analysis of inflammation-related indexes and blood cell counts for different outcomes. Systemic immune-inflammation index (SII), platelet-to-lymphocyte ratio (PLR), neutrophil-to-lymphocyte ratio (NLR), and the product of platelet count, neutrophil count (PPN), neutrophil count (NC), platelet (PLT) and lymphocyte count (LC). **(A–F)** represent clinical POPF, SSI, CD3 complications, hemorrhage, reoperation, and readmission, respectively.

The predictive power of the indicators was quantified by calculating the AUC from the ROC curve (**Figure 4**). SSI (AUC: 0.729, cutoff: 2314.608), NLR (AUC: 0.713, cutoff: 11.801), and NC (AUC: 0.706, cutoff: 10.635) were effective predictors of clinical POPF. The ability of PLR (AUC: 0.629, cutoff: 196.132) and PPN (AUC: 0.682, cutoff: 2501.940) to predict clinical POPF was relatively weak. PLR, PLT, and LC, on the other hand, were inadequate for predicting POPF. SII (AUC: 0.628, cutoff: 2240.490), NLR (AUC: 0.638, cutoff: 13.708), and NC (AUC: 0.624, cutoff: 8.295) were weak predictors of SSI. CD3 complications were poorly predicted by NLR (AUC: 0.606, cutoff: 11.332). LC was not a reliable predictor of readmission (AUC: 0.620, cutoff: 0.775). DCA analysis revealed the model provided clinical benefit across a wide range of threshold probabilities for predicting clinical POPF. However, for SSI and other outcomes, the threshold probability range conferring benefit was substantially narrower. The results of the DCA analysis are presented in **Figure 5**.

## Discussion

Despite being one of the most common tests conducted after PD, there has been limited systematic exploration of the association between CBC-related inflammatory indexes (including corresponding blood cell counts) and postoperative complications. Thus, we conducted the present study to identify associations between SII, NLR, PLR, PPN, NC, PLT, LC, and postoperative morbidity in PD patients. Our study revealed validated markers for different complications after PD, providing valuable insights for clinicians to interpret and utilize the results of blood routine tests following PD.

Routine tests, including CBC, are crucial tools for assessing the overall condition of a patient, particularly in the perioperative management of complex, high-morbidity procedures like PD. Compared to other indicators such as C-reactive protein, procalcitonin, cytokines, and immune cell subsets, complete blood count tests offer a simpler and more cost-effective means of obtaining important information without adding financial burden to the patient. Postoperative inflammation serves as an indicator of the risk of complications, and neutrophils, lymphocytes, and platelets are key cells associated with inflammation. Moreover, various inflammation-related indices have been proposed for further clinical application. In the context of patients undergoing surgical stress, the internal inflammatory state is altered compared to the general population. Determining which indicators are meaningful for evaluating outcomes in PD and identifying appropriate cutoff values are challenges that our clinical team has encountered.

SII, an indicator that combines NC, PLT, and LC, was initially introduced by Hu et al. and has been found to correlate with tumor prognosis (26). Previous studies have shown associations between SII and prognosis in patients with periampullary cancer, distal cholangiocarcinoma, and pancreatic head cancer undergoing PD surgery (18, 19, 27). In the present study, we found that SII was linked to several complications after PD, including clinical POPF and SSI, and possibly CD3 complications, although there was inconsistency between the main results and sensitivity analysis. In prior research, preoperative NLR emerged as a prognostic predictor in patients with distal bile duct cancer (28). NLR has attracted significant attention in the investigation of postoperative complications following PD compared to SII. Elevated preoperative NLR has been linked to comprehensive postoperative morbidity and hemorrhage (29, 30). Herein, we conducted a comprehensive analysis of specific outcomes and discovered that increased NLR was associated with clinical POPF, SSI, CD3 complications, and readmission. However, it should be borne in mind that in the RCS analysis of NLR with readmission, the p-value was 0.089. A related study also defined the threshold for the *P*-overall as < 0.10 (31). Similarly, PLR has been associated with postoperative prognosis after PD in patients with pancreatic head tumors (32). Our study suggested that PLR was linked to clinical POPF and might be related to CD3 complications, in contrast to a previous study that did not find a correlation between PLR and POPF (33). In our analysis, the evidence indicating that an increased PLR led to an increased risk of POPF was substantiated by logistic regression, RCS, and sensitivity analyses, bolstering our confidence in the association between PLR and POPF. On the other hand, the relationship between PPN and postoperative complications, as well as the prognosis of PD, had not been previously reported. Our findings indicated that, besides POPF, PPN might be potentially associated with postoperative hemorrhage. Interestingly, NC consistently emerged as a significant factor associated with clinical POPF. However, when exploring the relationship between NC and SSI, the results were not entirely consistent across analyses. Lower PLT levels were linked to an increased risk of hemorrhage, while lower LC levels represented a higher risk of both POPF and SSI.

In this study, ROC curves and DCA analyses suggested that inflammation-related indices and blood cell counts performed better in predicting postoperative complications of PD, especially in predicting clinical POPF. In contrast, RCS analysis reflected more meaningful indicators, with NLR standing out as an indicator associated with the most outcomes, including clinical POPF, SSI, CD3 complications, and readmission. The predictive ability of NLR for clinical POPF, SSI, and CD3 complications was not inferior to SII. Therefore, NLR should be recommended as a primary indicator for postoperative routine blood tests. The RCS also provided NLR cutoff values for each of these four outcomes: 19.66, 18.34, 17.96, and 16.90. Meanwhile, in ROC curve, the respective cutoff values were 11.80, 13.71, 11.33, and 9.99. Given the relatively weak predictive ability of individual indicators, RCS is recommended, especially for predicting SSI, CD3 complications, and readmission. Notably, the only indicators associated with postoperative hemorrhage were PPN and PLT. Because PLT was simpler than PPN, we favored PLT (cut-off value: 138.24). NLR and PLT also contained NC, LC and PLT, and we recommended NLR and PLT should be primary indicators in CDCs for observing after PD.

Nevertheless, there were several limitations to our study. Indeed, single-center studies carry the potential for bias. Furthermore, our analysis primarily focused on POD3 indicators, while clinical practice often encounters late and sudden changes in these indicators. Thus, our study may not adequately account for this heterogeneity. Additionally, we did not conduct an analysis of combined indicators, nor did we incorporate the results of other tests, such as liver function and drainage fluid amylase, which are considerations for our future research plans.

## Conclusion

Overall, our study systematically analyzed postoperative routine blood test results and uncovered associations between inflammation-related indices (SII, NLR, PLR, and PPN) and blood cell counts (NC, PLT, and LC) with various postoperative complications of PD. An elevated SII may indicate an increased risk of clinical POPF, SSI, and CD3 complications. A higher NLR may signify a greater risk of clinical POPF, SSI, CD3 complications, and may readmission. PLR may be associated with clinical POPF and CD3 complications. PPN was found to be associated with POPF and hemorrhage, while outcomes associated with NC included clinical POPF and SSI. Lower PLT levels were linked to a higher risk of hemorrhage, and lower LC levels were associated with a greater risk of both POPF and SSI. Considering simplicity and validity, we recommend NLR and PLT as the primary indicators for post-operative monitoring in routine blood tests following PD.

## Data availability statement

The datasets presented in this article are not readily available because Data used for this analysis cannot be made publicly available due to ethical restriction. Requests to access the datasets should be directed to Kezhen Zong, zongkezhen23@163.com.

## Ethics statement

The studies involving humans were approved by the ethics committee of The First Affiliated Hospital of Chongqing Medical University. The studies were conducted in accordance with the local legislation and institutional requirements. The ethics committee/institutional review board waived the requirement of written informed consent for participation from the participants or the participants’ legal guardians/next of kin because This is a retrospective observational study. We did not follow up with the patients and only obtained information from the electronic medical record system.

## Author contributions

MM: Writing – original draft, Conceptualization, Formal Analysis, Methodology. GL: Writing – original draft, Conceptualization, Formal Analysis, Methodology. BZ: Writing – review & editing. KL: Writing – review & editing. ZW: Writing – review & editing. LK: Writing – review & editing. MaL: Writing – review & editing. MiL: Writing – review & editing. CZ: Writing – review & editing. HY: Writing – review & editing. SW: Methodology, Project administration, Supervision, Validation, Writing – review & editing. ZH: Methodology, Project administration, Supervision, Validation, Writing – review & editing. KZ: Conceptualization, Formal Analysis, Methodology, Project administration, Supervision, Validation, Writing – review & editing.
