# Supplementary material for: Comprehensive analysis of the association between inflammation indexes and complications in patients undergoing pancreaticoduodenectomy
Source: Front Immunol. 2023 Nov 23;14:1303283. doi: 10.3389/fimmu.2023.1303283 (PMC10702568; doi:10.3389/fimmu.2023.1303283)

| Table S1 Association between inflammation-related indexes and blood cell counts with clinical POPF | | | | | | | | | | | |
| --- | --- | --- | --- | --- | --- | --- | --- | --- | --- | --- | --- |
| Indexes | Model1 | | |  | Model2 | | |  | Model3 | | |
|  | OR | 95% CI | p-value |  | OR | 95% CI | p-value |  | OR | 95% CI | p-value |
| **SII** |  |  |  |  |  |  |  |  |  |  |  |
| Q1 (ref) | —— | —— | —— |  | —— | —— | —— |  | —— | —— | —— |
| Q2 | 6.4 | 1.96, 28.9 | 0.005 |  | 5 | 1.43, 23.5 | 0.02 |  | 4.83 | 1.32, 23.6 | 0.028 |
| Q3 | 8.93 | 2.80, 39.9 | <0.001 |  | 7.23 | 2.12, 33.7 | 0.004 |  | 6.35 | 1.75, 30.9 | 0.009 |
| Q4 | 16 | 5.12, 70.7 | <0.001 |  | 15.3 | 4.46, 72.3 | <0.001 |  | 14.1 | 3.90, 69.5 | <0.001 |
| **NLR** |  |  |  |  |  |  |  |  |  |  |  |
| Q1 (ref) | —— | —— | —— |  | —— | —— | —— |  | —— | —— | —— |
| Q2 | 2.77 | 0.95, 9.23 | 0.074 |  | 2.73 | 0.89, 9.51 | 0.09 |  | 2.32 | 0.73, 8.26 | 0.2 |
| Q3 | 5.16 | 1.89, 16.7 | 0.003 |  | 4.81 | 1.66, 16.2 | 0.006 |  | 3.05 | 0.99, 10.7 | 0.062 |
| Q4 | 9.89 | 3.71, 31.6 | <0.001 |  | 9.56 | 3.41, 31.9 | <0.001 |  | 7.39 | 2.55, 25.1 | <0.001 |
| **PLR** |  |  |  |  |  |  |  |  |  |  |  |
| Q1 (ref) | —— | —— | —— |  | —— | —— | —— |  | —— | —— | —— |
| Q2 | 1.73 | 0.69, 4.53 | 0.2 |  | 1.99 | 0.74, 5.55 | 0.2 |  | 2.04 | 0.73, 5.96 | 0.2 |
| Q3 | 2.45 | 1.01, 6.27 | 0.052 |  | 2.83 | 1.06, 7.92 | 0.041 |  | 2.39 | 0.83, 7.16 | 0.11 |
| Q4 | 3.58 | 1.52, 9.04 | 0.005 |  | 5.9 | 2.14, 17.6 | <0.001 |  | 5.94 | 2.00, 19.0 | 0.002 |
| **PPN** |  |  |  |  |  |  |  |  |  |  |  |
| Q1 (ref) | —— | —— | —— |  | —— | —— | —— |  | —— | —— | —— |
| Q2 | 1.81 | 0.70, 4.94 | 0.2 |  | 1.38 | 0.50, 4.00 | 0.5 |  | 1.19 | 0.40, 3.68 | 0.8 |
| Q3 | 2.18 | 0.86, 5.88 | 0.11 |  | 1.84 | 0.65, 5.50 | 0.3 |  | 1.82 | 0.60, 5.74 | 0.3 |
| Q4 | 5.83 | 2.45, 15.3 | <0.001 |  | 4.14 | 1.44, 12.8 | 0.01 |  | 4.36 | 1.41, 14.5 | 0.012 |
| **NC** |  |  |  |  |  |  |  |  |  |  |  |
| Q1 (ref) | —— | —— | —— |  | —— | —— | —— |  | —— | —— | —— |
| Q2 | 2.76 | 1.01, 8.35 | 0.055 |  | 1.96 | 0.68, 6.18 | 0.2 |  | 1.65 | 0.54, 5.49 | 0.4 |
| Q3 | 2.76 | 1.01, 8.35 | 0.055 |  | 1.92 | 0.65, 6.22 | 0.2 |  | 1.55 | 0.50, 5.17 | 0.5 |
| Q4 | 9.29 | 3.66, 27.2 | <0.001 |  | 5.9 | 2.05, 19.1 | 0.002 |  | 4.81 | 1.58, 16.3 | 0.008 |
| **PLT** |  |  |  |  |  |  |  |  |  |  |  |
| Q1 (ref) | —— | —— | —— |  | —— | —— | —— |  | —— | —— | —— |
| Q2 | 1.39 | 0.56, 3.55 | 0.5 |  | 1.62 | 0.59, 4.59 | 0.3 |  | 1.61 | 0.55, 4.83 | 0.4 |
| Q3 | 3.15 | 1.36, 7.73 | 0.009 |  | 3.55 | 1.33, 10.1 | 0.014 |  | 3.47 | 1.25, 10.3 | 0.02 |
| Q4 | 2.16 | 0.91, 5.37 | 0.086 |  | 1.76 | 0.56, 5.68 | 0.3 |  | 2.36 | 0.68, 8.54 | 0.2 |
| **LC** |  |  |  |  |  |  |  |  |  |  |  |
| Q1 (ref) | —— | —— | —— |  | —— | —— | —— |  | —— | —— | —— |
| Q2 | 1.15 | 0.52, 2.53 | 0.7 |  | 0.8 | 0.33, 1.93 | 0.6 |  | 0.84 | 0.32, 2.18 | 0.7 |
| Q3 | 0.79 | 0.34, 1.79 | 0.6 |  | 0.61 | 0.24, 1.53 | 0.3 |  | 0.81 | 0.30, 2.19 | 0.7 |
| Q4 | 0.77 | 0.33, 1.75 | 0.5 |  | 0.33 | 0.12, 0.87 | 0.027 |  | 0.38 | 0.13, 1.09 | 0.076 |
| SII: systemic immune-inflammation index; NLR: neutrophil-to-lymphocyte ratio; PLR: platelet-to-lymphocyte ratio; PPN: the product of platelet count and neutrophil count; NC: neutrophil count; PLT: platelet; LC: platelet: lymphocyte count. OR: odds ratio; CI: confidence interval. | | | | | | | | | | | |

| Table S2 Association between inflammation-related indexes and blood cell counts with SSI | | | | | | | | | | | |
| --- | --- | --- | --- | --- | --- | --- | --- | --- | --- | --- | --- |
| Indexes | Model1 | | |  | Model2 | | |  | Model3 | | |
|  | OR | 95% CI | p-value |  | OR | 95% CI | p-value |  | OR | 95% CI | p-value |
| **SII** |  |  |  |  |  |  |  |  |  |  |  |
| Q1 (ref) | —— | —— | —— |  | —— | —— | —— |  | —— | —— | —— |
| Q2 | 3.37 | 1.19, 11.1 | 0.03 |  | 3.03 | 0.99, 10.6 | 0.062 |  | 3 | 0.95, 10.9 | 0.073 |
| Q3 | 3.37 | 1.19, 11.1 | 0.03 |  | 2.94 | 0.95, 10.3 | 0.071 |  | 2.42 | 0.75, 8.79 | 0.2 |
| Q4 | 4.04 | 1.45, 13.2 | 0.012 |  | 4.07 | 1.30, 14.6 | 0.021 |  | 3.53 | 1.05, 13.4 | 0.049 |
| **NLR** |  |  |  |  |  |  |  |  |  |  |  |
| Q1 (ref) | —— | —— | —— |  | —— | —— | —— |  | —— | —— | —— |
| Q2 | 1.15 | 0.41, 3.29 | 0.8 |  | 1.12 | 0.38, 3.35 | 0.8 |  | 0.96 | 0.31, 3.03 | >0.9 |
| Q3 | 1.63 | 0.62, 4.50 | 0.3 |  | 1.53 | 0.56, 4.39 | 0.4 |  | 1.13 | 0.37, 3.49 | 0.8 |
| Q4 | 3.29 | 1.35, 8.69 | 0.011 |  | 3.23 | 1.26, 8.95 | 0.018 |  | 2.87 | 1.02, 8.66 | 0.051 |
| **PLR** |  |  |  |  |  |  |  |  |  |  |  |
| Q1 (ref) | —— | —— | —— |  | —— | —— | —— |  | —— | —— | —— |
| Q2 | 1.81 | 0.70, 4.94 | 0.2 |  | 2.23 | 0.81, 6.43 | 0.12 |  | 2.37 | 0.84, 7.08 | 0.11 |
| Q3 | 2.18 | 0.86, 5.88 | 0.11 |  | 2.66 | 0.96, 7.82 | 0.065 |  | 2.33 | 0.79, 7.21 | 0.13 |
| Q4 | 1.81 | 0.70, 4.94 | 0.2 |  | 2.92 | 0.98, 9.15 | 0.058 |  | 3.23 | 1.0, 11.0 | 0.054 |
| **PPN** |  |  |  |  |  |  |  |  |  |  |  |
| Q1 (ref) | —— | —— | —— |  | —— | —— | —— |  | —— | —— | —— |
| Q2 | 1.3 | 0.47, 3.68 | 0.6 |  | 0.91 | 0.31, 2.71 | 0.9 |  | 0.83 | 0.27, 2.60 | 0.7 |
| Q3 | 2.59 | 1.04, 6.92 | 0.046 |  | 1.93 | 0.69, 5.67 | 0.2 |  | 1.89 | 0.65, 5.81 | 0.2 |
| Q4 | 1.99 | 0.78, 5.40 | 0.2 |  | 1.02 | 0.32, 3.28 | >0.9 |  | 0.93 | 0.28, 3.13 | >0.9 |
| **NC** |  |  |  |  |  |  |  |  |  |  |  |
| Q1 (ref) | —— | —— | —— |  | —— | —— | —— |  | —— | —— | —— |
| Q2 | 1.71 | 0.62, 4.98 | 0.3 |  | 1.52 | 0.52, 4.68 | 0.5 |  | 1.36 | 0.44, 4.40 | 0.6 |
| Q3 | 1.9 | 0.70, 5.49 | 0.2 |  | 1.52 | 0.52, 4.68 | 0.5 |  | 1.15 | 0.37, 3.74 | 0.8 |
| Q4 | 3.55 | 1.41, 9.87 | 0.01 |  | 2.46 | 0.84, 7.78 | 0.11 |  | 2.02 | 0.64, 6.75 | 0.2 |
| **PLT** |  |  |  |  |  |  |  |  |  |  |  |
| Q1 (ref) | —— | —— | —— |  | —— | —— | —— |  | —— | —— | —— |
| Q2 | 1.3 | 0.47, 3.68 | 0.6 |  | 1.16 | 0.39, 3.53 | 0.8 |  | 1.04 | 0.34, 3.26 | >0.9 |
| Q3 | 3.55 | 1.46, 9.35 | 0.007 |  | 3.13 | 1.14, 9.22 | 0.031 |  | 2.83 | 1.01, 8.48 | 0.053 |
| Q4 | 1.3 | 0.47, 3.68 | 0.6 |  | 0.77 | 0.21, 2.79 | 0.7 |  | 0.74 | 0.19, 2.89 | 0.7 |
| **LC** |  |  |  |  |  |  |  |  |  |  |  |
| Q1 (ref) | —— | —— | —— |  | —— | —— | —— |  | —— | —— | —— |
| Q2 | 1.19 | 0.50, 2.89 | 0.7 |  | 0.85 | 0.33, 2.22 | 0.7 |  | 0.88 | 0.31, 2.48 | 0.8 |
| Q3 | 1.02 | 0.41, 2.53 | >0.9 |  | 0.7 | 0.26, 1.89 | 0.5 |  | 0.75 | 0.24, 2.23 | 0.6 |
| Q4 | 0.9 | 0.36, 2.25 | 0.8 |  | 0.37 | 0.12, 1.06 | 0.069 |  | 0.31 | 0.09, 1.02 | 0.058 |
| SII: systemic immune-inflammation index; NLR: neutrophil-to-lymphocyte ratio; PLR: platelet-to-lymphocyte ratio; PPN: the product of platelet count and neutrophil count; NC: neutrophil count; PLT: platelet; LC: platelet: lymphocyte count. OR: odds ratio; CI: confidence interval. | | | | | | | | | | | |

| Table S3 Association between inflammation-related indexes and blood cell counts with CD3 complication | | | | | | | | | | | |
| --- | --- | --- | --- | --- | --- | --- | --- | --- | --- | --- | --- |
| Indexes | Model1 | | |  | Model2 | | |  | Model3 | | |
|  | OR | 95% CI | p-value |  | OR | 95% CI | p-value |  | OR | 95% CI | p-value |
| **SII** |  |  |  |  |  |  |  |  |  |  |  |
| Q1 (ref) | —— | —— | —— |  | —— | —— | —— |  | —— | —— | —— |
| Q2 | 1.27 | 0.48, 3.43 | 0.6 |  | 1.4 | 0.49, 4.12 | 0.5 |  | 1.33 | 0.44, 4.06 | 0.6 |
| Q3 | 0.75 | 0.25, 2.16 | 0.6 |  | 0.88 | 0.27, 2.77 | 0.8 |  | 0.88 | 0.26, 2.94 | 0.8 |
| Q4 | 1.42 | 0.55, 3.78 | 0.5 |  | 1.93 | 0.66, 5.78 | 0.2 |  | 2.11 | 0.68, 6.83 | 0.2 |
| **NLR** |  |  |  |  |  |  |  |  |  |  |  |
| Q1 (ref) | —— | —— | —— |  | —— | —— | —— |  | —— | —— | —— |
| Q2 | 1 | 0.32, 3.12 | >0.9 |  | 0.85 | 0.26, 2.75 | 0.8 |  | 0.76 | 0.22, 2.56 | 0.7 |
| Q3 | 1.9 | 0.70, 5.49 | 0.2 |  | 1.99 | 0.70, 5.98 | 0.2 |  | 1.97 | 0.65, 6.37 | 0.2 |
| Q4 | 2.1 | 0.79, 6.03 | 0.15 |  | 2.35 | 0.84, 7.06 | 0.11 |  | 2.59 | 0.86, 8.38 | 0.1 |
| **PLR** |  |  |  |  |  |  |  |  |  |  |  |
| Q1 (ref) | —— | —— | —— |  | —— | —— | —— |  | —— | —— | —— |
| Q2 | 0.49 | 0.16, 1.40 | 0.2 |  | 0.53 | 0.16, 1.57 | 0.3 |  | 0.51 | 0.15, 1.63 | 0.3 |
| Q3 | 0.89 | 0.34, 2.30 | 0.8 |  | 1.16 | 0.41, 3.28 | 0.8 |  | 1.36 | 0.45, 4.08 | 0.6 |
| Q4 | 1.11 | 0.45, 2.81 | 0.8 |  | 2 | 0.71, 5.77 | 0.2 |  | 3.02 | 0.97, 9.76 | 0.059 |
| **PPN** |  |  |  |  |  |  |  |  |  |  |  |
| Q1 (ref) | —— | —— | —— |  | —— | —— | —— |  | —— | —— | —— |
| Q2 | 1.42 | 0.55, 3.78 | 0.5 |  | 1.18 | 0.43, 3.35 | 0.7 |  | 0.98 | 0.34, 2.88 | >0.9 |
| Q3 | 1 | 0.36, 2.77 | >0.9 |  | 0.76 | 0.24, 2.34 | 0.6 |  | 0.72 | 0.22, 2.27 | 0.6 |
| Q4 | 1 | 0.36, 2.77 | >0.9 |  | 0.65 | 0.18, 2.27 | 0.5 |  | 0.62 | 0.17, 2.22 | 0.5 |
| **NC** |  |  |  |  |  |  |  |  |  |  |  |
| Q1 (ref) | —— | —— | —— |  | —— | —— | —— |  | —— | —— | —— |
| Q2 | 2.5 | 0.91, 7.63 | 0.086 |  | 2.73 | 0.90, 9.17 | 0.086 |  | 2.18 | 0.68, 7.67 | 0.2 |
| Q3 | 1.59 | 0.53, 5.06 | 0.4 |  | 1.8 | 0.55, 6.35 | 0.3 |  | 1.51 | 0.44, 5.51 | 0.5 |
| Q4 | 2.03 | 0.71, 6.29 | 0.2 |  | 1.93 | 0.56, 7.17 | 0.3 |  | 1.62 | 0.44, 6.37 | 0.5 |
| **PLT** |  |  |  |  |  |  |  |  |  |  |  |
| Q1 (ref) | —— | —— | —— |  | —— | —— | —— |  | —— | —— | —— |
| Q2 | 0.77 | 0.27, 2.11 | 0.6 |  | 0.75 | 0.25, 2.23 | 0.6 |  | 0.71 | 0.23, 2.18 | 0.6 |
| Q3 | 1.25 | 0.49, 3.24 | 0.6 |  | 1.24 | 0.43, 3.61 | 0.7 |  | 1.25 | 0.43, 3.72 | 0.7 |
| Q4 | 0.88 | 0.32, 2.37 | 0.8 |  | 0.79 | 0.21, 2.83 | 0.7 |  | 1 | 0.26, 3.80 | >0.9 |
| **LC** |  |  |  |  |  |  |  |  |  |  |  |
| Q1 (ref) | —— | —— | —— |  | —— | —— | —— |  | —— | —— | —— |
| Q2 | 0.69 | 0.26, 1.78 | 0.4 |  | 0.55 | 0.19, 1.50 | 0.2 |  | 0.52 | 0.18, 1.45 | 0.2 |
| Q3 | 0.82 | 0.32, 2.07 | 0.7 |  | 0.6 | 0.21, 1.64 | 0.3 |  | 0.5 | 0.16, 1.45 | 0.2 |
| Q4 | 0.61 | 0.22, 1.62 | 0.3 |  | 0.35 | 0.11, 1.04 | 0.064 |  | 0.26 | 0.07, 0.83 | 0.028 |
| SII: systemic immune-inflammation index; NLR: neutrophil-to-lymphocyte ratio; PLR: platelet-to-lymphocyte ratio; PPN: the product of platelet count and neutrophil count; NC: neutrophil count; PLT: platelet; LC: platelet: lymphocyte count. OR: odds ratio; CI: confidence interval. | | | | | | | | | | | |

| Table S4 Association between inflammation-related indexes and blood cell counts with hemorrhage | | | | | | | | | | | |
| --- | --- | --- | --- | --- | --- | --- | --- | --- | --- | --- | --- |
| Indexes | Model1 | | |  | Model2 | | |  | Model3 | | |
|  | OR | 95% CI | p-value |  | OR | 95% CI | p-value |  | OR | 95% CI | p-value |
| **SII** |  |  |  |  |  |  |  |  |  |  |  |
| Q1 (ref) | —— | —— | —— |  | —— | —— | —— |  | —— | —— | —— |
| Q2 | 1.63 | 0.62, 4.50 | 0.3 |  | 1.69 | 0.59, 5.13 | 0.3 |  | 1.77 | 0.58, 5.63 | 0.3 |
| Q3 | 0.59 | 0.17, 1.89 | 0.4 |  | 0.59 | 0.16, 2.05 | 0.4 |  | 0.59 | 0.15, 2.16 | 0.4 |
| Q4 | 1.15 | 0.41, 3.29 | 0.8 |  | 1.11 | 0.35, 3.57 | 0.9 |  | 1.38 | 0.41, 4.80 | 0.6 |
| **NLR** |  |  |  |  |  |  |  |  |  |  |  |
| Q1 (ref) | —— | —— | —— |  | —— | —— | —— |  | —— | —— | —— |
| Q2 | 2.16 | 0.64, 8.50 | 0.2 |  | 1.96 | 0.56, 7.88 | 0.3 |  | 1.98 | 0.55, 8.16 | 0.3 |
| Q3 | 3.9 | 1.28, 14.6 | 0.025 |  | 4.03 | 1.27, 15.6 | 0.026 |  | 4.97 | 1.44, 20.6 | 0.016 |
| Q4 | 2.48 | 0.76, 9.62 | 0.2 |  | 2.36 | 0.70, 9.42 | 0.2 |  | 3.12 | 0.87, 13.2 | 0.094 |
| **PLR** |  |  |  |  |  |  |  |  |  |  |  |
| Q1 (ref) | —— | —— | —— |  | —— | —— | —— |  | —— | —— | —— |
| Q2 | 0.59 | 0.20, 1.61 | 0.3 |  | 0.65 | 0.21, 1.87 | 0.4 |  | 0.63 | 0.20, 1.88 | 0.4 |
| Q3 | 0.78 | 0.29, 2.07 | 0.6 |  | 0.9 | 0.31, 2.58 | 0.8 |  | 0.97 | 0.32, 2.90 | >0.9 |
| Q4 | 0.59 | 0.20, 1.61 | 0.3 |  | 0.69 | 0.21, 2.13 | 0.5 |  | 1.04 | 0.30, 3.47 | >0.9 |
| **PPN** |  |  |  |  |  |  |  |  |  |  |  |
| Q1 (ref) | —— | —— | —— |  | —— | —— | —— |  | —— | —— | —— |
| Q2 | 0.48 | 0.17, 1.26 | 0.15 |  | 0.31 | 0.10, 0.89 | 0.035 |  | 0.24 | 0.07, 0.75 | 0.019 |
| Q3 | 0.48 | 0.17, 1.26 | 0.15 |  | 0.27 | 0.08, 0.84 | 0.027 |  | 0.3 | 0.09, 0.94 | 0.045 |
| Q4 | 0.48 | 0.17, 1.26 | 0.15 |  | 0.2 | 0.05, 0.69 | 0.014 |  | 0.22 | 0.06, 0.79 | 0.024 |
| **NC** |  |  |  |  |  |  |  |  |  |  |  |
| Q1 (ref) | —— | —— | —— |  | —— | —— | —— |  | —— | —— | —— |
| Q2 | 1.71 | 0.62, 4.98 | 0.3 |  | 1.35 | 0.45, 4.18 | 0.6 |  | 1.19 | 0.38, 3.91 | 0.8 |
| Q3 | 1.17 | 0.39, 3.56 | 0.8 |  | 0.88 | 0.27, 2.93 | 0.8 |  | 0.82 | 0.24, 2.84 | 0.8 |
| Q4 | 1.17 | 0.39, 3.56 | 0.8 |  | 0.71 | 0.20, 2.54 | 0.6 |  | 0.73 | 0.19, 2.78 | 0.6 |
| **PLT** |  |  |  |  |  |  |  |  |  |  |  |
| Q1 (ref) | —— | —— | —— |  | —— | —— | —— |  | —— | —— | —— |
| Q2 | 0.53 | 0.18, 1.42 | 0.2 |  | 0.47 | 0.15, 1.38 | 0.2 |  | 0.41 | 0.12, 1.25 | 0.13 |
| Q3 | 0.7 | 0.26, 1.82 | 0.5 |  | 0.58 | 0.19, 1.69 | 0.3 |  | 0.57 | 0.19, 1.71 | 0.3 |
| Q4 | 0.44 | 0.14, 1.23 | 0.13 |  | 0.26 | 0.06, 1.0 | 0.055 |  | 0.34 | 0.08, 1.35 | 0.14 |
| **LC** |  |  |  |  |  |  |  |  |  |  |  |
| Q1 (ref) | —— | —— | —— |  | —— | —— | —— |  | —— | —— | —— |
| Q2 | 0.75 | 0.27, 2.07 | 0.6 |  | 0.67 | 0.22, 1.94 | 0.5 |  | 0.66 | 0.21, 1.99 | 0.5 |
| Q3 | 1.15 | 0.44, 3.00 | 0.8 |  | 1.03 | 0.37, 2.84 | >0.9 |  | 0.86 | 0.29, 2.54 | 0.8 |
| Q4 | 0.45 | 0.13, 1.37 | 0.2 |  | 0.28 | 0.07, 0.96 | 0.05 |  | 0.19 | 0.04, 0.70 | 0.017 |
| SII: systemic immune-inflammation index; NLR: neutrophil-to-lymphocyte ratio; PLR: platelet-to-lymphocyte ratio; PPN: the product of platelet count and neutrophil count; NC: neutrophil count; PLT: platelet; LC: platelet: lymphocyte count. OR: odds ratio; CI: confidence interval. | | | | | | | | | | | |

| Table S5 Association between inflammation-related indexes and blood cell counts with reoperation | | | | | | | | | | | |
| --- | --- | --- | --- | --- | --- | --- | --- | --- | --- | --- | --- |
| Indexes | Model1 | | |  | Model2 | | |  | Model3 | | |
|  | OR | 95% CI | p-value |  | OR | 95% CI | p-value |  | OR | 95% CI | p-value |
| **SII** |  |  |  |  |  |  |  |  |  |  |  |
| Q1 (ref) | —— | —— | —— |  | —— | —— | —— |  | —— | —— | —— |
| Q2 | 1 | 0.26, 3.79 | >0.9 |  | 0.85 | 0.20, 3.58 | 0.8 |  | 0.84 | 0.19, 3.60 | 0.8 |
| Q3 | 0.58 | 0.11, 2.47 | 0.5 |  | 0.52 | 0.09, 2.46 | 0.4 |  | 0.49 | 0.08, 2.59 | 0.4 |
| Q4 | 1 | 0.26, 3.79 | >0.9 |  | 0.98 | 0.22, 4.23 | >0.9 |  | 1 | 0.21, 4.71 | >0.9 |
| **NLR** |  |  |  |  |  |  |  |  |  |  |  |
| Q1 (ref) | —— | —— | —— |  | —— | —— | —— |  | —— | —— | —— |
| Q2 | 0.79 | 0.19, 3.12 | 0.7 |  | 0.73 | 0.16, 3.02 | 0.7 |  | 0.61 | 0.13, 2.74 | 0.5 |
| Q3 | 0.79 | 0.19, 3.12 | 0.7 |  | 0.77 | 0.17, 3.21 | 0.7 |  | 0.86 | 0.17, 4.22 | 0.8 |
| Q4 | 1 | 0.26, 3.79 | >0.9 |  | 0.94 | 0.24, 3.71 | >0.9 |  | 0.88 | 0.20, 3.73 | 0.9 |
| **PLR** |  |  |  |  |  |  |  |  |  |  |  |
| Q1 (ref) | —— | —— | —— |  | —— | —— | —— |  | —— | —— | —— |
| Q2 | 0.15 | 0.01, 0.93 | 0.086 |  | 0.16 | 0.01, 1.02 | 0.1 |  | 0.19 | 0.01, 1.30 | 0.15 |
| Q3 | 0.82 | 0.22, 2.88 | 0.8 |  | 0.92 | 0.23, 3.61 | >0.9 |  | 0.9 | 0.20, 3.91 | 0.9 |
| Q4 | 1 | 0.29, 3.39 | >0.9 |  | 1.25 | 0.32, 4.84 | 0.7 |  | 1.82 | 0.42, 8.03 | 0.4 |
| **PPN** |  |  |  |  |  |  |  |  |  |  |  |
| Q1 (ref) | —— | —— | —— |  | —— | —— | —— |  | —— | —— | —— |
| Q2 | 1.45 | 0.44, 5.19 | 0.5 |  | 1.07 | 0.29, 4.13 | >0.9 |  | 0.94 | 0.24, 3.83 | >0.9 |
| Q3 | 0.38 | 0.05, 1.84 | 0.3 |  | 0.29 | 0.04, 1.55 | 0.2 |  | 0.27 | 0.03, 1.62 | 0.2 |
| Q4 | 0.79 | 0.19, 3.12 | 0.7 |  | 0.42 | 0.07, 2.22 | 0.3 |  | 0.32 | 0.05, 1.79 | 0.2 |
| **NC** |  |  |  |  |  |  |  |  |  |  |  |
| Q1 (ref) | —— | —— | —— |  | —— | —— | —— |  | —— | —— | —— |
| Q2 | 2.16 | 0.64, 8.50 | 0.2 |  | 1.43 | 0.39, 6.00 | 0.6 |  | 0.87 | 0.21, 3.91 | 0.8 |
| Q3 | 0.48 | 0.06, 2.58 | 0.4 |  | 0.35 | 0.04, 2.06 | 0.3 |  | 0.25 | 0.03, 1.55 | 0.2 |
| Q4 | 1 | 0.23, 4.43 | >0.9 |  | 0.6 | 0.11, 3.16 | 0.5 |  | 0.35 | 0.05, 2.07 | 0.2 |
| **PLT** |  |  |  |  |  |  |  |  |  |  |  |
| Q1 (ref) | —— | —— | —— |  | —— | —— | —— |  | —— | —— | —— |
| Q2 | 1 | 0.23, 4.43 | >0.9 |  | 1.13 | 0.23, 5.52 | 0.9 |  | 1.33 | 0.26, 6.98 | 0.7 |
| Q3 | 1.27 | 0.32, 5.39 | 0.7 |  | 1.3 | 0.29, 6.27 | 0.7 |  | 1.66 | 0.34, 8.87 | 0.5 |
| Q4 | 1.27 | 0.32, 5.39 | 0.7 |  | 0.99 | 0.17, 5.99 | >0.9 |  | 1.03 | 0.16, 6.93 | >0.9 |
| **LC** |  |  |  |  |  |  |  |  |  |  |  |
| Q1 (ref) | —— | —— | —— |  | —— | —— | —— |  | —— | —— | —— |
| Q2 | 1.2 | 0.34, 4.39 | 0.8 |  | 1.19 | 0.31, 4.69 | 0.8 |  | 1.26 | 0.31, 5.44 | 0.7 |
| Q3 | 0.8 | 0.19, 3.18 | 0.7 |  | 0.71 | 0.15, 3.00 | 0.6 |  | 0.71 | 0.14, 3.32 | 0.7 |
| Q4 | 0.58 | 0.11, 2.47 | 0.5 |  | 0.31 | 0.05, 1.57 | 0.2 |  | 0.18 | 0.02, 1.07 | 0.072 |
| SII: systemic immune-inflammation index; NLR: neutrophil-to-lymphocyte ratio; PLR: platelet-to-lymphocyte ratio; PPN: the product of platelet count and neutrophil count; NC: neutrophil count; PLT: platelet; LC: platelet: lymphocyte count. OR: odds ratio; CI: confidence interval. | | | | | | | | | | | |

| Table S6 Association between inflammation-related indexes and blood cell counts with readmission | | | | | | | | | | | |
| --- | --- | --- | --- | --- | --- | --- | --- | --- | --- | --- | --- |
| Indexes | Model1 | | |  | Model2 | | |  | Model3 | | |
|  | OR | 95% CI | p-value |  | OR | 95% CI | p-value |  | OR | 95% CI | p-value |
| **SII** |  |  |  |  |  |  |  |  |  |  |  |
| Q1 (ref) | —— | —— | —— |  | —— | —— | —— |  | —— | —— | —— |
| Q2 | 2.16 | 0.64, 8.50 | 0.2 |  | 1.8 | 0.48, 7.67 | 0.4 |  | 1.65 | 0.41, 7.49 | 0.5 |
| Q3 | 0.74 | 0.14, 3.49 | 0.7 |  | 0.62 | 0.11, 3.23 | 0.6 |  | 0.63 | 0.11, 3.44 | 0.6 |
| Q4 | 1.27 | 0.32, 5.39 | 0.7 |  | 1.24 | 0.27, 5.86 | 0.8 |  | 1.27 | 0.26, 6.55 | 0.8 |
| **NLR** |  |  |  |  |  |  |  |  |  |  |  |
| Q1 (ref) | —— | —— | —— |  | —— | —— | —— |  | —— | —— | —— |
| Q2 | 1 | 0.26, 3.79 | >0.9 |  | 0.87 | 0.22, 3.36 | 0.8 |  | 0.59 | 0.13, 2.49 | 0.5 |
| Q3 | 0.79 | 0.19, 3.12 | 0.7 |  | 0.68 | 0.15, 2.82 | 0.6 |  | 0.42 | 0.08, 2.00 | 0.3 |
| Q4 | 1.22 | 0.35, 4.48 | 0.8 |  | 1.02 | 0.28, 3.84 | >0.9 |  | 0.85 | 0.20, 3.55 | 0.8 |
| **PLR** |  |  |  |  |  |  |  |  |  |  |  |
| Q1 (ref) | —— | —— | —— |  | —— | —— | —— |  | —— | —— | —— |
| Q2 | 1.56 | 0.42, 6.39 | 0.5 |  | 1.43 | 0.37, 6.05 | 0.6 |  | 1.24 | 0.30, 5.55 | 0.8 |
| Q3 | 1 | 0.23, 4.43 | >0.9 |  | 1.12 | 0.24, 5.26 | 0.9 |  | 1.3 | 0.26, 6.58 | 0.7 |
| Q4 | 1.56 | 0.42, 6.39 | 0.5 |  | 2.13 | 0.51, 9.79 | 0.3 |  | 3.05 | 0.64, 15.8 | 0.2 |
| **PPN** |  |  |  |  |  |  |  |  |  |  |  |
| Q1 (ref) | —— | —— | —— |  | —— | —— | —— |  | —— | —— | —— |
| Q2 | 1 | 0.29, 3.39 | >0.9 |  | 0.77 | 0.21, 2.78 | 0.7 |  | 0.67 | 0.16, 2.61 | 0.6 |
| Q3 | 0.82 | 0.22, 2.88 | 0.8 |  | 0.52 | 0.12, 2.11 | 0.4 |  | 0.56 | 0.12, 2.34 | 0.4 |
| Q4 | 0.47 | 0.10, 1.89 | 0.3 |  | 0.25 | 0.04, 1.28 | 0.11 |  | 0.28 | 0.04, 1.45 | 0.14 |
| **NC** |  |  |  |  |  |  |  |  |  |  |  |
| Q1 (ref) | —— | —— | —— |  | —— | —— | —— |  | —— | —— | —— |
| Q2 | 1 | 0.29, 3.39 | >0.9 |  | 0.83 | 0.23, 3.05 | 0.8 |  | 0.67 | 0.16, 2.66 | 0.6 |
| Q3 | 0.64 | 0.16, 2.38 | 0.5 |  | 0.42 | 0.09, 1.69 | 0.2 |  | 0.4 | 0.09, 1.69 | 0.2 |
| Q4 | 0.64 | 0.16, 2.38 | 0.5 |  | 0.32 | 0.06, 1.44 | 0.15 |  | 0.28 | 0.05, 1.35 | 0.12 |
| **PLT** |  |  |  |  |  |  |  |  |  |  |  |
| Q1 (ref) | —— | —— | —— |  | —— | —— | —— |  | —— | —— | —— |
| Q2 | 1.85 | 0.53, 7.43 | 0.3 |  | 1.82 | 0.48, 7.87 | 0.4 |  | 1.71 | 0.42, 7.63 | 0.5 |
| Q3 | 1.85 | 0.53, 7.43 | 0.3 |  | 1.61 | 0.39, 7.42 | 0.5 |  | 1.67 | 0.40, 7.80 | 0.5 |
| Q4 | 0.48 | 0.06, 2.58 | 0.4 |  | 0.43 | 0.05, 3.09 | 0.4 |  | 0.58 | 0.06, 4.58 | 0.6 |
| **LC** |  |  |  |  |  |  |  |  |  |  |  |
| Q1 (ref) | —— | —— | —— |  | —— | —— | —— |  | —— | —— | —— |
| Q2 | 0.82 | 0.25, 2.65 | 0.7 |  | 0.73 | 0.21, 2.51 | 0.6 |  | 0.72 | 0.20, 2.57 | 0.6 |
| Q3 | 0.55 | 0.14, 1.93 | 0.4 |  | 0.53 | 0.12, 1.96 | 0.4 |  | 0.37 | 0.07, 1.56 | 0.2 |
| Q4 | 0.4 | 0.08, 1.51 | 0.2 |  | 0.33 | 0.06, 1.42 | 0.2 |  | 0.28 | 0.05, 1.38 | 0.14 |
| SII: systemic immune-inflammation index; NLR: neutrophil-to-lymphocyte ratio; PLR: platelet-to-lymphocyte ratio; PPN: the product of platelet count and neutrophil count; NC: neutrophil count; PLT: platelet; LC: platelet: lymphocyte count. OR: odds ratio; CI: confidence interval. | | | | | | | | | | | |

Figure S1 Visualization of the relationship between inflammatory index and outcome by RCS


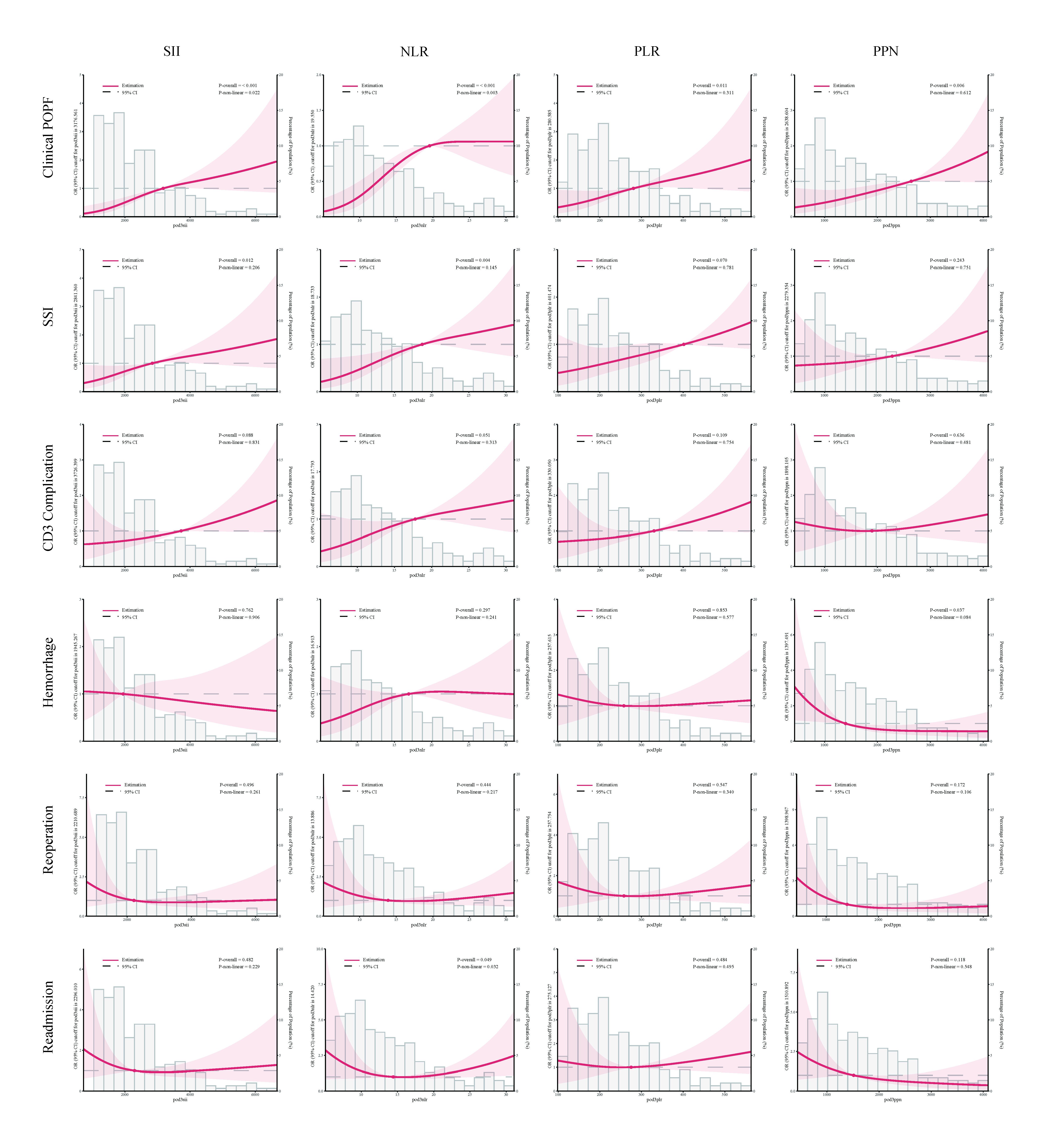


Figure S2 Visualization of the relationship between blood cell counts and outcome by RCS


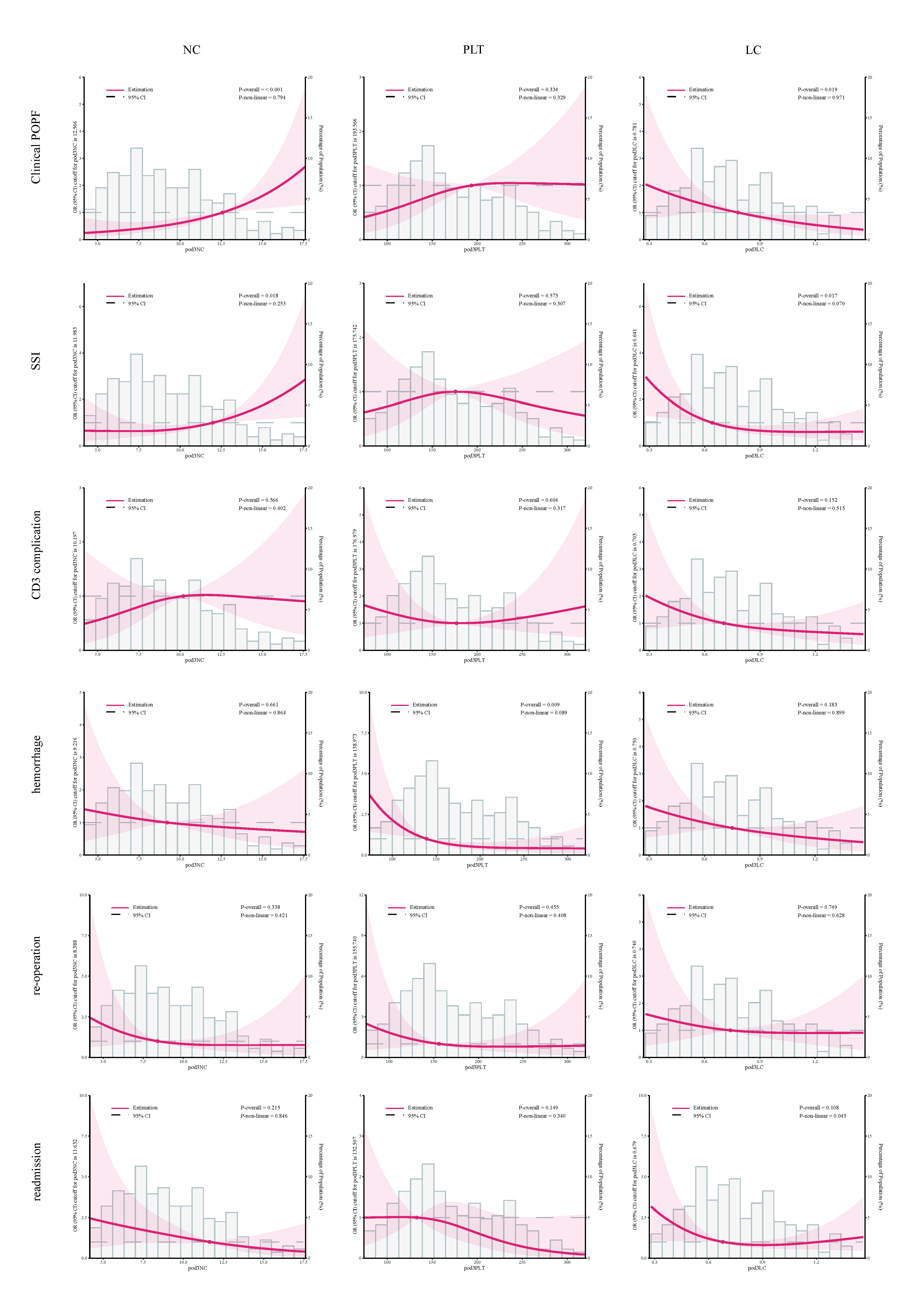

Supplement: Supplementary file 1 [file DataSheet_1.docx]
